# Electronic cigarette aerosols alter the expression of cisplatin transporters and increase drug resistance in oral cancer cells

**DOI:** 10.1038/s41598-021-81148-0

**Published:** 2021-01-19

**Authors:** Jimmy Manyanga, Vengatesh Ganapathy, Célia Bouharati, Toral Mehta, Balaji Sadhasivam, Pawan Acharya, Daniel Zhao, Lurdes Queimado

**Affiliations:** 1 Department of Otolaryngology Head and Neck Surgery, The University of Oklahoma Health Sciences Center, 800 Research Parkway, Room 431, Oklahoma City, OK 73104 USA; 2 Department of Cell Biology, The University of Oklahoma Health Sciences Center, Oklahoma City, OK USA; 3 Department of Biostatistics and Epidemiology, The University of Oklahoma Health Sciences Center, Oklahoma City, OK USA; 4 TSET Health Promotion Research Center, Stephenson Cancer Center, The University of Oklahoma Health Sciences Center, Oklahoma City, OK USA

**Keywords:** Head and neck cancer, Oral cancer, Cancer therapeutic resistance, Chemotherapy, Transport carrier

## Abstract

Tobacco smoking is the leading preventable cause of cancer. Moreover, continued smoking during cancer therapy reduces overall survival. Aware of the negative consequences of tobacco smoking and the challenges of smoking cessation, cancer patients are inquiring whether they should switch to electronic cigarettes (e-cigarettes). To obtain evidence-based data to inform this decision, we examined the effects of e-cigarette aerosol exposure on cisplatin resistance in head and neck cancer cells. Our results show that cancer cells exposed to e-cigarette aerosol extracts and treated with cisplatin have a significant decrease in cell death, increase in viability, and increase in clonogenic survival when compared to non-exposed cells. Moreover, exposure to e-cigarette aerosol extracts increased the concentration of cisplatin needed to induce a 50% reduction in cell growth (IC50) in a nicotine-independent manner. Tobacco smoke extracts induced similar increases in cisplatin resistance. Changes in the expression of drug influx and efflux transporters, rather than activation of cell growth-promoting pathways or DNA damage repair, contribute to e-cigarette induced cisplatin resistance. These results suggest that like combustible tobacco, e-cigarette use might increase chemotherapy resistance, and emphasize the urgent need for rigorous evaluation of e-cigarettes health effects to ensure evidence-based public health policies.

## Introduction

Head and neck squamous cell carcinoma (HNSCC) is the sixth most common malignancy, with over 657,000 newly diagnosed cases and 330,000 deaths per year worldwide^1^. Despite improved treatment options, including targeted therapies, the prognosis of HNSCC patients remains poor. The average 5-year survival rate has remained practically unchanged over the last 40 years: at 75–83% for patients with localized HNSCC, 45–65% for those whose cancer spread to regional lymph nodes, and only 33–40% for those with distant metastasis^2^. Only 29% of oral cavity and pharynx cancers are diagnosed at the local stage^2^. Thus, most head and neck tumors present as advanced disease, requiring combined modality approaches that frequently include radiotherapy and platinum-based chemotherapy, with or without surgery^3,4^. Platinum-based chemotherapy consisting of either cisplatin or carboplatin is the usual first-line treatment for inoperable recurrent or metastatic HNSCC^5^. In addition to late diagnosis, therapy resistance is one of the main factors contributing to poor cancer prognosis. Cisplatin resistance can emerge due to a host of environmental factors as well as genetic or epigenetic alterations in cancer cells^6^.

Tobacco smoking is the leading preventable cause of death and the main risk factor for lung and head and neck cancers. It is also well established that cigarette smoking has negative implications on cancer outcomes, including increasing disease progression, incidence of second primary tumors, and treatment-related toxicity, and reducing patient survival^7–12^. Smoking at the time of diagnosis has been shown to increase resistance to radio- and chemo-therapy^7,13^ and tumor recurrence^9,11,14,15^. Alarmingly, data show that about 60% of lung and head and neck cancer patients, who were cigarette smokers before their diagnosis, continued to smoke during treatment^16^. Concerned about the negative consequences of smoking but unable or unwilling to quit due to nicotine addiction, cancer patients, and consumers in general, are increasingly seeking other methods of nicotine delivery.

Electronic cigarettes (e-cigarettes) are battery-operated devices that heat a solution of chemicals (propylene glycol, glycerol, and flavorings) with or without nicotine, to produce an inhalable aerosol. Studies have shown that e-cigarette aerosols contain harmful and potentially harmful toxicants such as volatile organic compounds (e.g., acrolein, acetaldehyde, formaldehyde, benzaldehyde, acetone, and toluene)^17–19^, free-radicals^20^, and heavy metals (e.g., copper, zinc, chromium, iron, lead, cadmium, arsenic, aluminum, tin, and nickel)^21–25^. Some e-cigarette aerosol components are carcinogenic or potentially carcinogenic (e.g., formaldehyde and cadmium)^26^. Curiously, some of the individual constituents present in e-cigarette aerosols have been shown to increase chemotherapy sensitivity (e.g., glycerol)^27^, while others have been shown to increase chemotherapy resistance [e.g., nicotine and reactive oxygen species (ROS)]^28,29^. Yet, thanks to few restrictions and a lack of regulatory guidelines, e-cigarettes are aggressively marketed both as a safer alternative to combustible tobacco and as a smoking cessation aid. Consequently, the use of e-cigarettes has increased exponentially since introduction into the U.S. market in 2007. More than 5.3 million American high and middle school students reported using e-cigs in 2019^30^. Among adults, current smokers had the highest rate of use, with 52.5% reporting to ever used an e-cigarette and 10.8% currently using e-cigarettes^31^. The rate of e-cigarette use reported in lung and head and neck cancer patients was about 33% in 2013^32^, and is currently the highest (~ 80%) among cancer patients who are younger than 50 years old and current smokers^33^. These alarming numbers and the presence of harmful and potentially harmful chemicals in e-cigarette aerosols^24^, coupled with the paucity of short- and long-term studies on the safety of e-cigarettes, all stress the urgent need to investigate the potential effects of e-cigarettes on cancer treatment thoroughly.

E-cigarettes can deliver nicotine levels similar to those observed in smokers^34^. Nicotine, a main component of e-cigarette aerosol, has been shown to reduce cisplatin-induced cell death^28^. Other toxicants present in e-cigarette aerosols have also been shown to positively or negatively impact chemotherapy sensitivity. Hence, we hypothesized that exposure to e-cigarette aerosol might alter cisplatin resistance. Here we show that exposure of oral cancer cells to e-cigarette aerosol extracts increases cisplatin resistance through nicotine-dependent and independent mechanisms.

## Results

### Exposure to e-cigarette aerosol extracts reduces cancer cell death induced by cisplatin

**Figure 1:**
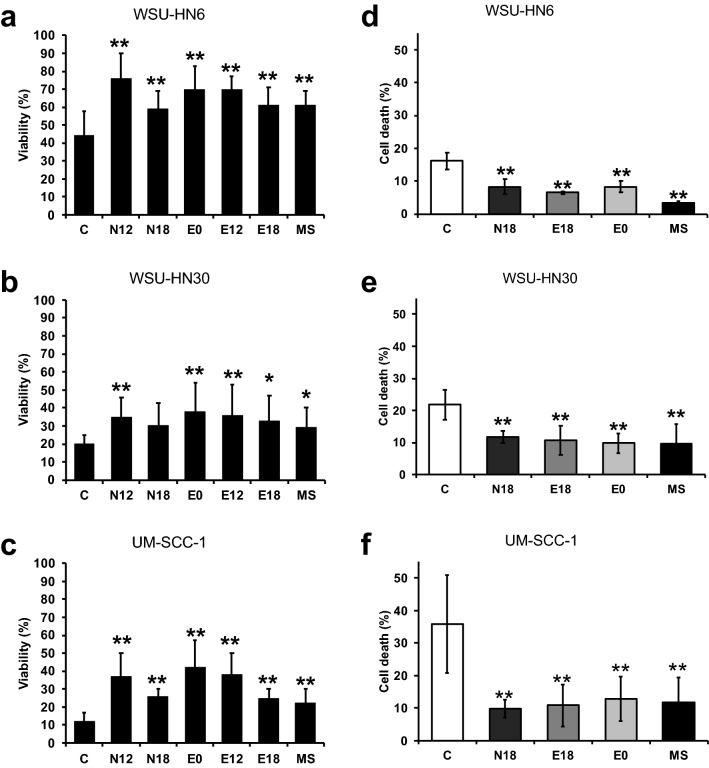
Exposure to e-cigarette aerosol extracts reduces cisplatin-induced cell death and increases cell viability. WSU-HN6, WSU-HN30, and UM-SCC-1 cell lines were exposed to e-cigarette aerosol extracts for 48 h, followed by 48 h treatment with cisplatin in the presence of e-cigarette extracts. (**a–c**) Fraction of viable cells after 48 h cisplatin treatment. Viability was assessed by the MTT test and normalized to non-cisplatin treatment. (**d–f**) Percentage of dead cells after 48 h cisplatin treatment. *p < 0.05, **p < 0.01 by One-way ANOVA.

Concurrent smoking during cancer therapy has been reported to increase chemo and/or radiotherapy resistance^7,13^. Moreover*, *in vitro studies have demonstrated that cigarette smoke condensate and nicotine can increase cancer therapy resistance in diverse cancer cell lines, including those derived from HNSCC^28,35^. Hence, it is essential to determine whether exposure to e-cigarette aerosols might modify cancer response to chemotherapy. To explore this important clinical question, we began by evaluating the effect of e-cigarette exposure on cell viability after cisplatin treatment. Three distinct HNSCC cell lines (UM-SSC-1, WSU-HN6, and WSU-HN30) were exposed to e-cigarette aerosol extracts for 48 h, followed by 48 h of treatment with cisplatin (10 µM) in the presence of e-cigarette aerosol extracts. Nicotine is the only tobacco smoke component with a well-documented role in cisplatin resistance^28^. To dissect the contribution of nicotine, within the complex e-cigarette aerosol mixture, to cisplatin resistance our study design included an extract without nicotine (E0). In total, we used five different e-cigarette extracts (N12, N18, E0, E12 and E18) obtained from two distinct brands of e-cigarettes with varying e-liquid nicotine content (0, 12 or 18 mg/ml). All extracts were diluted to a dose of 10 puffs/5 L, yielding exposures that encompass nicotine concentrations between 0 and 39 ng/ml (Table S1), a range similar to that observed in the plasma of vapers^34^. For comparison, cancer cells were exposed to a similar dose of mainstream tobacco smoke (MS) extracts. Cell viability was determined by MTT (3-[4,5-dimethylthiazol-2-yl]-2,5-diphenyltetrazolium bromide) assay. Exposure to e-cigarette aerosol extracts led to a significant increase in cell viability after cisplatin treatment (*p* < 0.01) in WSU-HN6 and UM-SCC-1 cells for all extracts compared to the vehicle control (Fig. 1a,c). In WSU-HN30 cells, all e-cigarette extracts except N18 caused a significant (*p* < 0.05) increase in cell viability after cisplatin treatment (Fig. 1b). Importantly, exposure to nicotine-free extracts (E0) also led to a significant increase in cell survival after cisplatin treatment (*p* < 0.01) in all cell lines, indicating that nicotine-independent mechanisms contribute to the observed results (Fig. 1a–c). We also observed a comparable increase in cell viability after cisplatin treatment in cells exposed to MS smoke extracts (Fig. 1a–c). Strengthening the significance of our findings, linear regression analysis adjusting for cell line showed that exposure to each of the e-cigarette extracts led to a significant (*p* < 0.001) increase in cell viability across all cell lines after cisplatin treatment.

To rule out the possibility that the observed increase in cell viability reflected an effect of the extracts alone on cell growth, we exposed UM-SCC-1, WSU-HN6, and WSU-HN30 cells to e-cigarette aerosol extracts alone for 96 h and measured cell proliferation by MTT assay. Consistent with previous reports^36^, exposure to e-cigarette aerosol or tobacco smoke extracts alone, at the doses used in this study, did not increase cell growth (Figure S1a–c). These findings suggest that the increase in cell viability observed after cisplatin treatment is not due to an increase in cell proliferation but instead to an increase in cisplatin resistance induced by the presence of e-cigarette aerosol extracts. To further understand the relationship between e-cigarette exposure, cisplatin treatment, and cell viability, we assessed the impact of e-cigarette aerosol extracts on cell death induced by cisplatin using the trypan blue exclusion method. Consistent with the cell viability results (Fig. 1a–c), we observed that exposure to e-cigarette aerosol extracts significantly (*p* < 0.01) reduced the cell death induced by cisplatin in all cell lines and for all extracts (Fig. 1d–f). The observed reduction in cell death was similar for e-cigarette extracts with and without nicotine (Fig. 1d–f). Linear regression analyses documented a significant (*p* < 0.0001) reduction in cisplatin-induced cell death for each e-cigarette aerosol exposure across all cell lines. Collectively, these data show that in vitro exposure to e-cigarette aerosol does not change cell proliferation but significantly reduces cell death induced by cisplatin. Remarkably, our data suggest that nicotine-independent mechanisms play a key role in the observed decrease in cisplatin-induced cell death.

### Exposure to e-cigarette aerosol extracts increases the dose of cisplatin required to induce a 50% reduction in cell growth (IC50)

**Figure 2:**
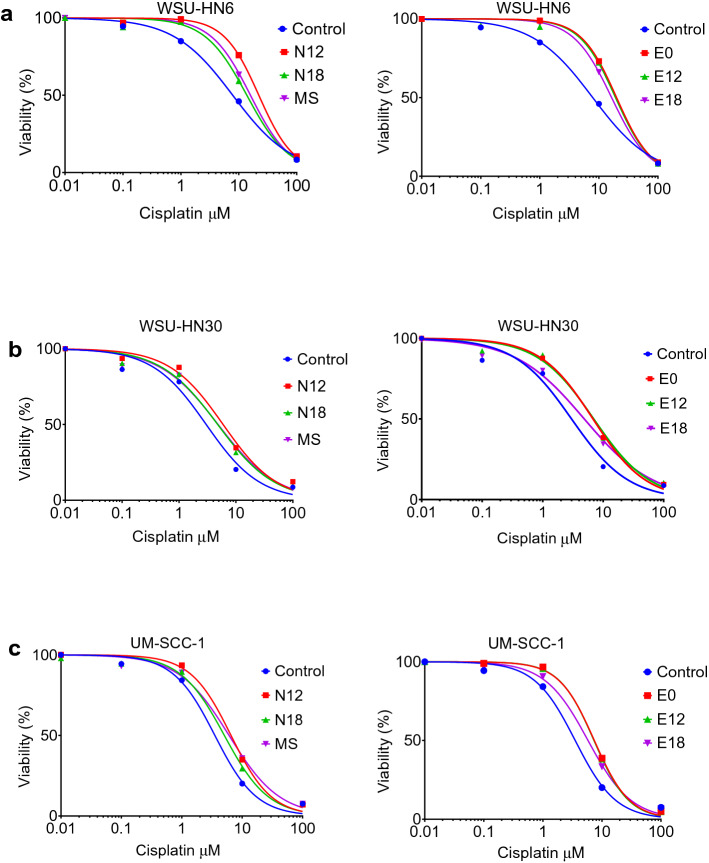
Exposure to e-cigarette aerosol extracts increases IC50. Dose–response curves after exposure to e-cigarette aerosol or MS extracts and 0.01–100 µM cisplatin for WSU-HN6 (**a**), WSU-HN30 (**b**), and UM-SCC-1 (**c**) cell lines. The dose of cisplatin required to induce a 50% reduction in cell growth (the half-maximal inhibitory concentration, IC50) was computed from fitting a symmetrical sigmoidal curve to the logarithm of the response with the logarithm of the cisplatin concentration as the predictor using GraphPad Prism software.

The determination of the concentrations of a drug needed to induce a 50% reduction in cell growth (the half-maximal inhibitory concentration or IC50) is an in vitro measure essential for understanding the pharmacological potency of a drug under specific conditions^37^, and has been reported to be predictive of cancer chemoresistance in patients^38^. Hence, to further investigate the potential clinical significance of our observations, we examined whether the presence of e-cigarette aerosol extracts altered the dose of cisplatin needed to induce a 50% reduction in head and neck cancer cell growth. HNSCC cells were exposed to e-cigarette aerosol extracts for 48 h and treated for an additional 48 h with extracts and cisplatin (0.01–100 μM). Compared to vehicle-exposed cells (control), exposure to e-cigarette aerosol extracts significantly increased the cisplatin IC50 values for all extracts and cell lines (Fig. 2a–c; Table 1). Consistent with the data shown in Fig. 1, there was no significant difference in cisplatin IC50 values between e-cigarette extracts delivering distinct nicotine concentrations (Table 1). This suggests once again that the cisplatin dose–response effect on cell viability is independent of the amount of nicotine in the e-cigarette extracts. Likewise, the increase in IC50 observed in cells exposed to e-cigarette aerosol extracts was comparable to that observed in cells exposed to MS smoke extracts (Fig. 2a–c; Table 1). Overall, these results suggest that like tobacco smoke, exposure to e-cigarette aerosol increases the resistance of cancer cells to cisplatin. Moreover, it suggests that mechanisms independent of nicotine contribute to the cisplatin resistance induced by e-cigarettes.

**Table 1:** Cisplatin IC50 values calculated for exposure and each cell line.

	WSU-HN6		WSU-HN30		UM-SCC-1	
	IC50 [95% CI] μM	*p* Value	IC50 [95% CI] μM	*p* Value	IC50 [95% CI] μM	*p* Value
Control	7.9 [6.2 to 10.0]	1	3.0 [2.4 to 3.8]	1	3.5 [2.9 to 4.3]	1
N12	22.2 [19.0 to 26.0]	< 0.0001	5.9 [4.2 to 8.4]	0.0008	6.5 [5.3 to 8.0]	< 0.0001
N18	13.8 [12.1 to 15.8]	< 0.0001	4.8 [3.6 to 6.4]	0.013	5.1 [4.2 to 6.0]	0.0071
E0	20.0 [17.7 to 22.5]	< 0.0001	6.8 [4.9 to 9.4]	< 0.0001	7.4 [6.4 to 8.6]	< 0.0001
E12	19.1 [16.8 to 21.8]	0.0002	7.0 [4.7 to 10.4]	0.0002	7.3 [6.2 to 6.4]	< 0.0001
E18	16.4 [14.3 to 19.0]	0.0004	4.9 [3.4 to 7.0]	0.021	5.8 [5.0 to 6.7]	< 0.0001
MS	15.9 [13.3 to 19.3]	< 0.0001	4.9 [4.0 to 5.9]	0.002	6.1 [4.9 to 7.4]	0.0003

*p Values shown compared to the vehicle-exposed (control) cells.

### Exposure to e-cigarette aerosol during cisplatin treatment increases clonogenic survival

**Figure 3:**
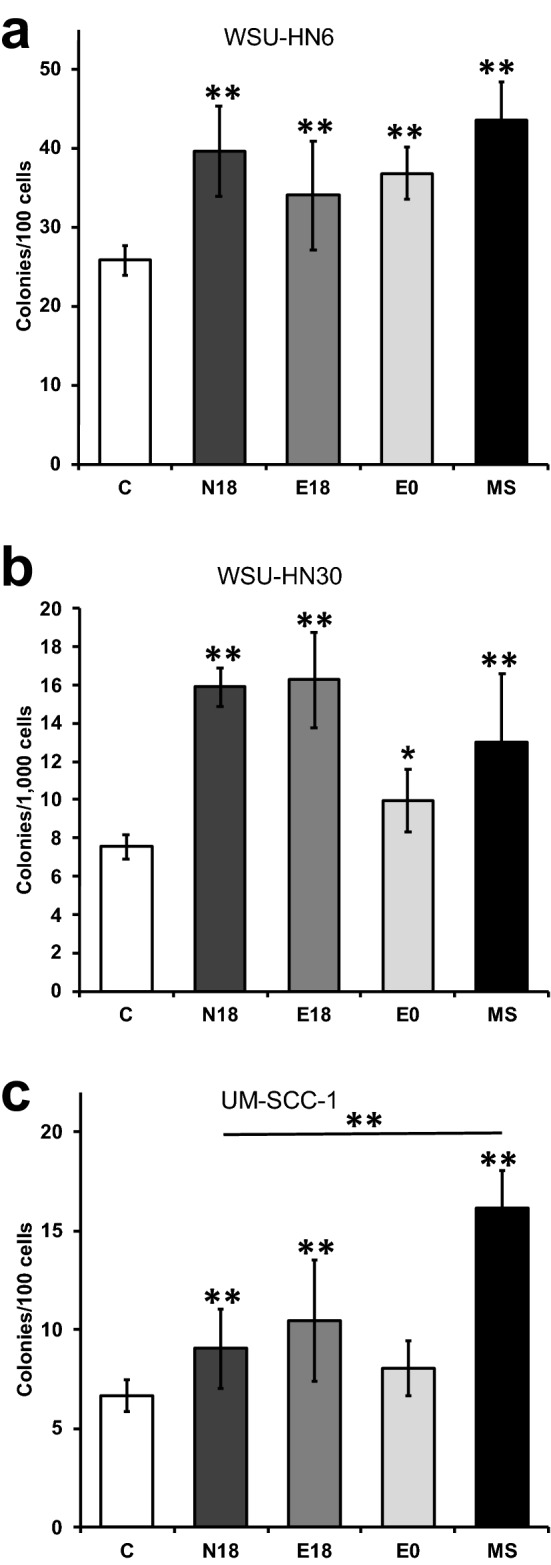
Exposure to e-cigarette aerosol extracts increases clonogenic survival after cisplatin. Quantification of colony survival fraction from WSU-HN6 (**a**), WSU-HN30 (**b**), and UM-SCC-1 (**c**) cells treated with extracts and cisplatin. Colonies were stained with 0.5% crystal violet and counted. Data presented as the number of colonies formed per number of cells plated. Crossbar denotes a significant difference between MS treated cells and all three e-cigarette extracts. *p < 0.05, **p < 0.001 by One-way ANOVA.

Chemotherapy resistance in HNSCC patients is a challenge and often leads to cancer progression, recurrence, and metastasis^39^. Cell viability after cisplatin treatment provides a measure of cisplatin effects on a short time interval (2 days; approximately 3–6 somatic generations), but does not inform about the indefinite reproductive viability of the surviving cells^40^. To further elucidate the potential clinical implications of e-cigarette use during cisplatin treatment, we used the clonogenic survival assay to test whether cancer cells surviving cisplatin treatment retained their indefinite reproductive ability. Cells were exposed to e-cigarette aerosol extracts for 48 h, followed by 48 h treatment with cisplatin in the presence of e-cigarette aerosol extracts. Cells were then collected and plated for clonogenic survival assessment. The formation of large colonies visible by eye after 1–2 weeks (Figure S2) indicates that the cells have survived cisplatin treatment and have retained the ability to reproduce indefinitely. As shown in Fig. 3a–c, treatment with cisplatin in the presence of e-cigarette aerosol extracts yielded significantly higher clonal survival in all cell lines and for all extracts tested. These data strengthen the hypothesis that e-cigarette use might increase cisplatin resistance. The number of WSU-HN6 and WSU-HN30 cells surviving cisplatin treatment and maintaining indefinite reproductive viability were comparable between e-cigarette aerosol and MS smoke extract-exposed cells (Fig. 3a,b). However, UM-SCC-1 cells exposed to MS smoke extracts showed a significantly (*p* < 0.01) higher increase in clonogenic survival after cisplatin treatment than cells exposed to e-cigarette extracts (Fig. 3c). These differences might reflect cell line-specific responses to tobacco smoke. Notably, WSU-HN6 cells exposed to e-cigarette extracts with or without nicotine and treated with cisplatin formed similar number of colonies (Fig. 3a), pinpointing a key role of nicotine-independent mechanisms in clonogenic survival. For cell lines WSU-HN30 and UM-SCC-1, the increase in clonogenic cell survival after cisplatin treatment was also considerable for e-cigarette extracts lacking nicotine (E0), although smaller than for e-cigarette extracts containing nicotine (Fig. 3b,c). These data show that, for some cancer cell lines, the presence of nicotine in the e-cigarette aerosol increases the number of cancer cells that retain the ability to reproduce indefinitely after cisplatin treatment. Thus, overall our data shows that both nicotine and non-nicotine mechanisms play a role in the observed increase in clonogenic survival after cisplatin treatment induced by e-cigarette aerosol extracts.

### Exposure to e-cigarette aerosol reduces *MMS19* and *ERCC1* expression

**Figure 4:**
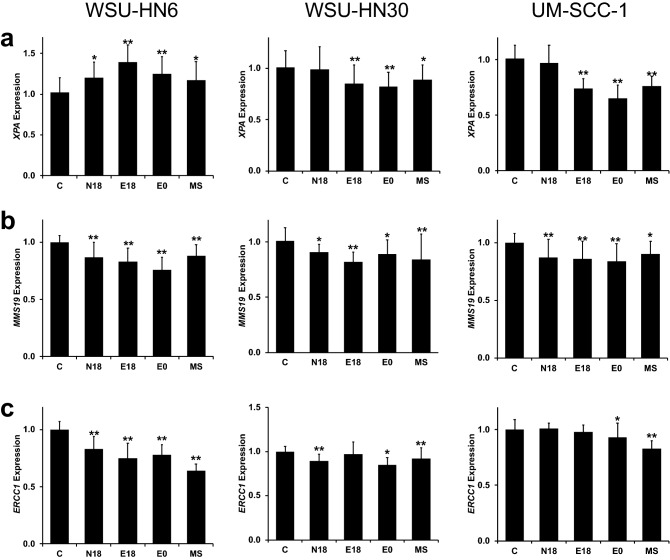
Exposure to e-cigarette aerosol extracts decreases the expression of DNA damage repair genes. (**a**) *XPA*, (**b**) *MMS19*, and (**c**) *ERCC1* mRNA expression from cells exposed to e-cigarette extracts alone for 48 h was assessed by qRT-PCR. *p < 0.05, **p < 0.001 by one-way ANOVA.

The principal mechanism of cisplatin cytotoxicity is the formation of platinum–DNA adducts. Thus cellular mechanisms leading to a reduction in cisplatin-induced DNA damage are key factors in the development of cisplatin resistance^41^. The majority of cisplatin-induced DNA lesions, intra-strand adducts, and inter-strand crosslinks are removed by nucleotide excision repair. Importantly, we and others have previously reported that long-term exposure to e-cigarette aerosol leads to a decrease in DNA repair capacity in non-cancer cells, due to a decrease in the expression of base excision and nucleotide excision repair enzymes^36,42,43^. Thus, we investigated whether under the current exposure conditions e-cigarette aerosol alters the expression of *XPA, MMS19*, and *ERCC1*, genes essential for the removal of cisplatin-induced DNA damage. After 48 h of exposure, the effect of e-cigarette aerosol and MS smoke extracts on *XPA* mRNA expression varied across cell lines (Fig. 4a). In contrast, *MMS19* mRNA expression was considerably reduced in all three cell lines exposed to e-cigarette extracts compared to vehicle-exposed cells (Fig. 4b). The decrease in *MMS19* expression was significant (*p* < 0.01) in WSU-HN6 and UM-SCC-1 for all e-cigarette aerosol extracts, and in WSU-HN30 for E18 (Fig. 4a). *ERCC1* mRNA expression was also significantly reduced by exposure to e-cigarette extracts in WSU-HN6 and WSU-HN30 cells (Fig. 4c). The observed decreases in *MMS19* and *ERCC1* expression were similar after exposure to e-cigarette extracts with or without nicotine (E0), and thus are nicotine-independent (Fig. 4a–c). After exposure to MS smoke, we observed a significant (*p* < 0.05) reduction in *MMS19* and *ERCC1* expression in all three cell lines (Fig. 4b,c). Together with the previously reported decrease in nucleotide excision repair, both in vitro and in vivo*,* after long-term exposure to e-cigarette aerosol^36,42,43^, our data strongly suggest that other mechanisms, rather than an increase in DNA damage repair, contribute to the observed increase in cisplatin resistance.

### E-cigarette aerosol exposure alters the expression of drug influx and efflux transporters

**Figure 5:**
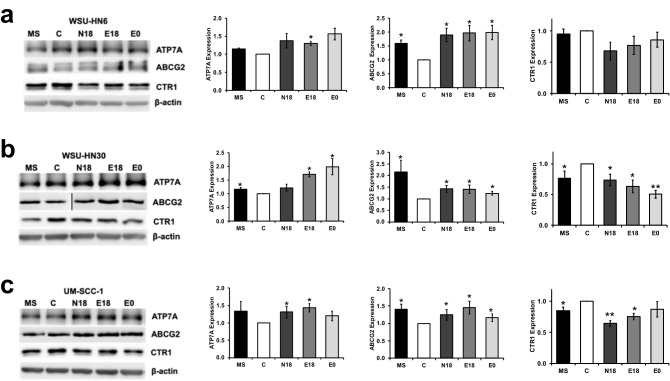
Exposure to e-cigarette aerosol extracts alters the expression of multidrug drug resistance proteins. (**a**) WSU-HN6; (**b**) WSU-HN30; (**c**) UM-SCC-1. Western blots are shown on the left and the quantification for each protein is shown on the right. Data shows that exposure to e-cigarette aerosol extracts decreased CTR1 and increased ABCG2 and ATP7A expression in all cell lines. Values shown as mean ± SEM, *p < 0.05, **p < 0.01 by unpaired two-tailed Student t-test. Vertical line delineates lane re-arrangement for the ABCG2 blot.

In addition to faster repairing of DNA lesions, reduced cisplatin accumulation inside cancer cells is one of the primary mechanisms responsible for cisplatin resistance^44^. The reduced cisplatin accumulation associated with cisplatin resistance is primarily due to reduced drug uptake rather than to increased drug efflux^44^. Moreover, the bulk of cisplatin is actively moved in and out of the cell by copper transporters (CTR1, ATP7A and ATP7B)^44^. Nonetheless, the following ATP-binding cassette (ABC) ATPase genes *ABCG2, ABCC2, ABCA1*, and *ABCC1* have been associated with cisplatin resistance in diverse cancers^41,44–47^ Thus, we investigated the mRNA expression levels of *CTR1*, *ATP7A*, *ATP7B*, *ABCG2, ABCC2, ABCA1*, and *ABCC1* after exposure to e-cigarette extracts alone without cisplatin. Following 48 h of e-cigarette exposure, we observed that the expression of the copper transporter *CTR1*, which mediates the uptake of cisplatin, was down-regulated at the mRNA level in WSU-HN6 for all e-cigarette extracts tested, but remained unchanged in the other cell lines (Figure S3a). The copper-extruding P-type ATPase Alpha, *ATP7A*, was substantially up-regulated in all three cell lines after exposure to e-cigarette aerosol extracts compared to vehicle-control cells. The increase in *ATP7A* expression reached significance (*p* < 0.05) for all e-cigarette extracts in WSU-HN6 and UM-SCC-1 (Figure S3b). Among the other drug efflux ATPase genes previously reported to contribute to cisplatin resistance *ABCG2, ABCC2, ABCA1*, and *ABCC1* were significantly up-regulated in at least one cell line (Figure S3c–f), while *ATP7B* was unchanged after exposure to e-cigarette aerosol extract (Figure S3g). Of these, ABCG2 extrudes the major tobacco carcinogen benzo(a)pyrene and its conjugates, and has been identified as the main ABC gene contributing to tobacco smoke-associated cisplatin resistance^35^. Thus, based on our findings and ABCG2 known role in tobacco-associated cisplatin resistance, CTR1, ATP7A and ABCG2 transporters were selected for further study. We observed that exposure to e-cigarette aerosol extracts led to a significant (*p* < 0.05) increase in ABCG2 protein expression in all cell lines and for all extracts (Fig. 5). Consistent with the literature^35^, we also observed a significant increase in ABCG2 expression in all cell lines after exposure to MS smoke. The observed increase in ABCG2 after exposure to e-cigarette aerosols was similar to that induced by MS smoke for WSU-HN6 and UM-SCC-1, but slightly lower for WSU-HN30 (*p* < 0.05). A considerable increase in ATP7A and a decrease in CTR1 protein expression following exposure to e-cigarette aerosol were also observed for all cell lines tested (Fig. 5a–c). The increase in ATP7A was significant (*p* < 0.05) for all cell lines following exposure to E18. The decrease in CTR1 expression was significant for all extracts in cell line WSU-HN30 (Fig. 5b). The alterations in the expression of membrane transporters induced by e-cigarette aerosol were similar for extracts with and without nicotine. Once again, these observations pinpoint the prominence of a nicotine-independent mechanism leading to changes in cisplatin transporters protein expression for all cell lines. Linear regression analyses showed that, when adjusted for cell lines, exposure to each of the e-cigarette aerosol extracts significantly decreased CTR1 (*p* < 0.001) and increased ATP7A (*p* < 0.05) and ABCG2 (*p* ≤ 0.001) expression. Our data clearly shows that exposure to e-cigarette aerosol alters the expression of the main known cisplatin active membrane transporters, which might lead to reduced cisplatin influx and increased efflux and consequently increase in cisplatin resistance. Supporting the clinical relevance of these findings, the expression of CTR1, ATP7A and ABCG2 has been reported to have predictive value in the response to platinum-based chemotherapy and patient prognosis in tobacco-associated cancers^48–50^.

## Discussion

We have uncovered a novel effect of e-cigarette aerosol, the induction of cisplatin resistance in cancer cells, through nicotine-dependent and -independent mechanisms. To our knowledge, this is the first report documenting that e-cigarette aerosol has the potential to negatively impact cancer treatment and ultimately patient survival. This is highly relevant as e-cigarettes are used by over 10% of all cancer patients^33^, 33% of head and neck cancer patients^32^, and 80% of cancer patients younger than 50 years old and current smokers^33^. Moreover, nearly 50% of all cancer patients, and the vast majority of head and neck cancer patients, are treated with cisplatin^44^. In the present study we investigate whether exposure to e-cigarette aerosol alters cisplatin response in cancer cell lines established from oral and pharyngeal mucosa, a primary site for e-cigarette aerosol deposition^51^. Our data show that, at least in vitro, e-cigarette exposure during cisplatin treatment reduces cancer cell death and increases the dose of cisplatin required to induce a 50% reduction in cell growth (IC50) in head and neck cancer cells. Consistent with previous reports^35^, we also observed that exposure to tobacco smoke extracts increased cisplatin resistance in oral cancer cell lines. These data are clinically relevant as in vitro cisplatin resistance has been reported to be predictive of cancer chemoresistance in patients^38^, and resistance to platinum-based chemotherapy is one of the main factors contributing to poor patient prognosis^48^.

Exposure to e-cigarette aerosol significantly increased cisplatin resistance in all HNSCC cell lines tested, but the amplitude of the increase varied by cell line (Table 1). Notably, the impact of extract exposure on cisplatin-induced cell death and cisplatin resistance was similar for both e-cigarette aerosol and tobacco smoke. These findings suggest that both combustible tobacco and e-cigarette use have similar potential to induce cisplatin resistance among HNSCC tumors. Patient data showing that continued cigarette smoking after a cancer diagnosis cuts the survival rate by 50%, in part by reducing the efficacy of cancer treatments^7,13,52^, further supports the clinical relevance of these in vitro studies.

Unlimited replicative potential is a key hallmark of cancer. We showed that exposing HNSCC cells to e-cigarette extracts during cisplatin treatment significantly increased clonogenic survival, a marker of unlimited reproductive viability of the surviving cells^40^. Curiously, while the effect of e-cigarette exposure on cisplatin-induced cell death and cell growth (IC50) was independent of nicotine content, the amplitude of the increase in clonogenic survival, although still considerable, was lower in the absence of nicotine in two (UM-SCC-1 and UM-HN30) of the cell lines studied (Fig. 3). These data suggest that upon exposure to e-cigarette aerosol, both nicotine-dependent and nicotine-independent mechanisms contribute to the observed unlimited replicative potential of the cisplatin surviving cells. These data are highly relevant as clonogenic survival is a well-known marker of future responsiveness to cancer therapy^53^.

Nicotine, a main component of e-cigarette aerosol, has been reported to increase cisplatin resistance in oral, lung, and bladder cell lines by inhibiting cisplatin-induced apoptosis^28,54–56^. Nicotine has also been shown to significantly increase tumor cell proliferation in bladder and oral cancer cells^55,56^. In contrast, we observed that exposure to e-cigarette aerosol did not increase cell proliferation (Figure S1), and both nicotine-containing and nicotine-free e-cigarette extracts induced similar changes in cisplatin-induced cell death and IC50 (Figs. 1, 2). We have also previously reported that exposure of oral and lung cells to e-cigarette aerosol extracts delivering up to 39 ng/ml of nicotine does not increase cell growth^36^. Several possibilities can account for these differences. First of all, our studies delivered a complex aerosol mixture containing a dose of nicotine (0–39 ng/ml) similar to that commonly observed in the plasma of vapers and smokers^34^, while the studies cited above used pure nicotine at much higher nicotine concentrations (146–1460 ng/ml). So, it is possible that the proliferative and antiapoptotic effects of nicotine are only seen at much higher nicotine doses. Supporting this hypothesis, a dose- and time-dependent effect of nicotine on oral cancer cell survival has recently been reported, with low nicotine concentration (15 ng/ml) showing no effect, and 146 ng/ml increasing cancer cell survival and reducing cisplatin-induced apoptosis^56^. Another possibility is that some e-cigarette aerosol components counteracted the proliferative and anti-apoptotic effects of nicotine. In favor of this hypothesis, glycerol a main e-cigarette humectant, constituting 30–70% of most e-liquids, has been shown to inhibit oral cancer cell growth and enhance cisplatin-induced apoptosis^27^. Altogether, these data show that at the clinically relevant nicotine concentration used in our study, nicotine is not the major contributor to the observed increase in cisplatin resistance associated with e-cigarette aerosol exposure. Nonetheless, our data also shows that the presence of nicotine in the e-cigarette aerosol can increase the number of cancer cells that retain their indefinite reproductive ability (Fig. 3b,c). Thus, for two of the HNSCC cell lines tested, clonogenic survival after cisplatin was the highest in the presence of nicotine, suggesting that nicotine role in cisplatin resistance might vary across tumor cells. One of these cell lines (UM-SCC-1), established from a smoker, also showed a higher increase in clonogenic survival in MS smoke than in e-cigarette aerosol exposed cells. This observation suggests that, although harmful, the use of e-cigarettes during cisplatin treatment may be less detrimental than the use of combustible cigarettes for some patients.

Collectively, our data suggest that nicotine-independent mechanisms play a significant role in the observed increase in cisplatin resistance. Nicotine-independent mechanisms have been reported in several e-cigarette health effects, including induction of oxidative stress and DNA damage in oral cell lines^36^, disruption of lung lipid homeostasis and innate immunity in mice^57^, and impairment of vascular reactivity and endothelial function in healthy nonsmokers^58^. Potential candidates for the observed nicotine-independent cisplatin resistance include e-cigarette flavorings, humectant propylene glycol, ROS, and metals (e.g., copper, zinc and arsenic) present in the aerosol. Flavorings and flavored e-liquids without nicotine have been shown to trigger inflammatory responses, mediated by ROS production, *in vitro*^59^. E-cigarette aerosols containing propylene glycol with and without nicotine have also been shown to induce the release of cytokines such as IL-6, type I interferon (IFN) and other pro-inflammatory mediators^60^. Metals present in the aerosol (e.g., copper, zinc and cobalt) have been shown to modulate drug transport at a posttranscriptional level^61^, as well as to contribute to increased ROS levels^62^. In turn, ROS and pro-inflammatory mediators such as IL-6 and IFN have been reported to increase chemotherapy resistance by up-regulating anti-apoptotic genes, stimulating stemness, increasing repair of damaged DNA, and increasing drug efflux^29,41,63^. Exposure to metals (e.g., copper, zinc, cadmium), oxidative stress, or inflammatory cytokines (e.g., IL-6) can also increase metallothioneins^64^, a class of low-molecular-weight thiol-containing proteins that bind and inactivate cisplatin and is considered one of the most important mechanisms of cisplatin resistance^44^. Consistent with these mechanisms being physiologically relevant, e-cigarette users have been shown to have increased levels of systemic biomarkers of oxidative stress^65^ and inflammation (e.g., IL-1β, IL-6, IL-8, IL-13, IFN-γ, and MMP-9)^66^, and increased levels of metals and metallothioneins in the urine^67^. Overall, these findings show that besides nicotine, e-cigarette users are exposed to significant levels of ROS, metals and other aerosol components which can potentially contribute to cisplatin resistance by inducing multiple non-redundant molecular or cellular events, including inflammatory responses and oxidative stress, increasing drug detoxification, and altering cisplatin drug transporters.

The anti-tumor function of cisplatin, an intercalating agent, has been linked to its ability to crosslink with the purine bases on the DNA, causing DNA damage, interfering with DNA repair mechanisms, and subsequently inducing cell cycle arrest and apoptosis in cancer cells^46^. Yet, cisplatin resistance stems from complex genetic and epigenetic changes occurring in almost every mechanism supporting cell survival, including cell growth-promoting pathways, DNA damage repair, cell-surface binding, and transporters for cisplatin^47^. As discussed above, at the dose of e-cigarette tested, no increase in cell growth was observed. Our data also show that short-term exposure (48 h) to e-cigarette extract causes a decrease in the expression of nucleotide excision repair genes essential for the removal of cisplatin-induced DNA lesions. Supporting the relevance of these findings, long-term exposure to e-cigarette aerosols consistently^42^ leads to a decrease in the expression of DNA repair genes (*ERCC1*, *XPC*, *OGG1*, *ATM*) *in vitro*^36^, in mice^43^, and in e-cigarette users^68^. These data document that the increase in cisplatin resistance observed during e-cigarette aerosol exposure is not due to cell growth-promoting pathways or to an increase in DNA damage repair.

Copper transporters are the principal efflux and influx transmembrane transportation system for cisplatin^41,45^. Despite, not having the dominant role in cisplatin resistance, ABC proteins, such as ABCG2, ABCC2, ABCA1, and ABCC1, transport a wide variety of chemical compounds in an ATP-dependent manner and play an important role in cisplatin resistance in diverse cancers^41,45–47^. Of these, ABCG2 has been characterized as the main ABC protein contributing to tobacco smoke-associated cisplatin resistance^35^. Consistent with the multifactorial nature of cisplatin resistance^41^, our data show that exposure to e-cigarette aerosol extracts significantly alters the expression of the main cisplatin drug transporters (CTR1, ATP7A, and ABCG2) in all cell lines studied. We also observed a significant increase in *ABCC2, ABCA1*, and *ABCC1* mRNA, in at least one cell line, following exposure to all e-cigarette extracts. At the protein level, our linear regression analysis also consistently showed that, when adjusted for cell lines, exposure to each of the e-cigarette aerosol extracts significantly decreased the expression of CTR1 (*p* < 0.001), the principal active cisplatin influx transporter, and increased the expression of multidrug efflux transporters ATP7A (*p* < 0.05) and ABCG2 (*p* ≤ 0.001). The alterations in protein expression were nicotine-independent, as they were observed even after exposure to the nicotine-free e-cigarette aerosol extract (Fig. 5). Similar alterations were also present after exposure to tobacco smoke extracts. These alterations can lead to a decrease in cisplatin cellular accumulation, one of the most common causes of cisplatin resistance^47^. Supporting, the relevance of our findings, clinical studies have demonstrated that CTR1 expression correlates with the intra-tumoral concentration of cisplatin and patient outcomes following cisplatin therapy^61^. For example, low CTR1 expression, as well as high ATP7A expression, are associated with poor platinum-based chemotherapy outcomes^49,50^, while high CTR1 expression correlates with longer progression free survival and overall survival after platinum-based regimens^61,69^. Additionally, in head and neck cancer stem cells cisplatin resistance can be modulated by RXRα through CTR1 expression^70^. An increase in ABCG2 expression has also been observed in over 50% of head and neck squamous cell carcinomas^48^. ABCG2 is considered a marker of cancer stem cells and its expression levels in head and neck cancer tissues correlate with the patients’ smoking history^35^. Moreover, in vitro, exposure to tobacco smoke condensate up-regulates ABCG2 in head and neck cancer cell lines leading to an increase in cisplatin resistance^35^. Altogether, these data suggest that e-cigarette exposure reduces cisplatin uptake and retention, thereby decreasing the formation of platinum–DNA adducts and consequently reducing cisplatin cytotoxicity.

While changes in protein expression for cisplatin transporters were consistent across cell lines, at the mRNA level, the effect of e-cigarette aerosol in the expression of cisplatin transporters varied by cell line, with for example *ATP7A* showing an increase in all cell lines tested and *ABCG2*, *ABCC1,* and *ABCC2* showing variable expression across cell lines. These mRNA alterations probably reflect the intrinsic genetic and epigenetic heterogeneity among the different cancer cell lines tested. Most importantly, these data suggest that the cisplatin resistance observed is most probably due to an impact of e-cigarette aerosol exposure on post-transcriptional mechanisms, rather than on transcriptional mechanisms. Metals in e-cigarette aerosols could contribute to post-transcriptional alterations in cisplatin transporters. Copper can reach levels 6 times higher in e-cigarette aerosol than those reported for conventional cigarette smoke^71^, and has been shown to change CTR1 and ATP7A protein levels by post-transcriptional regulatory mechanisms^72,73^. In intestinal cells, an increase in copper leads to an increase in ATP7A protein stability^72^. In yeast cells, exposure to copper leads to the degradation and internalization of CTR1, decreasing the cellular accumulation of cisplatin and increasing cisplatin resistance^73^. Zinc and cobalt have also been shown to regulate CTR1 at the post-transcriptional level^61^. Redox signaling networks have also been shown to regulate the expression of multidrug transporters, specifically ABCG2, on multiple levels including transcriptional, translational, post-translational, and epigenetic levels^29^. In summary, several e-cigarette aerosol components can potentially affect cisplatin resistance. Follow up studies identifying the precise mechanism by which diverse e-cigarette aerosol components, individually or combined, might contribute to the observed cisplatin resistance are essential to guide FDA regulations and improve public health. Regardless, our work paves the way for additional studies addressing e-cigarette use among cancer patients and therapy outcomes, which are critical to identify the potential risks associated with e-cigarette use and to guide much-needed e-cigarette regulation.

Taken together, to the best of our knowledge, our study is the first to show that e-cigarette aerosol exposure, at doses experience by users, increases cisplatin resistance in vitro to the same measure as tobacco smoke exposure. Our data also suggests that, rather than nicotine, other aerosol components might lead to post-translational changes in the main cisplatin drug transporters leading to cisplatin resistance. Understanding and targeting these potential mechanisms might improve cisplatin effectiveness. Our findings have major clinical relevance as they suggest that, like tobacco smoking, e-cigarette use during chemotherapy might increase cisplatin resistance, reducing therapy efficacy, and worsening patient prognosis.

## Material and methods

### Cell culture

Human epithelial cancer cell lines from different head and neck regions UM-SCC-1 (floor of mouth), WSU-HN6 (tongue), and WSU-HN30 (pharyngeal) were cultured in Dulbecco’s modified Eagle’s Medium (DMEM) supplemented with 10% fetal bovine serum, 1% L-glutamine, and 1% antibiotic–antimycotic (Gibco). Cell cultures were maintained in an incubator at 37 °C and 5% CO_2._ All cell lines used were verified and authenticated using short tandem repeats (STR)-based DNA profiling and multiplex PCR by CellCheck Cell Line Authentication (IDEXX BioResearch, Missouri, USA).

### E-cigarette aerosol extracts, tobacco smoke extracts, and cisplatin

E-cigarette [NJOY (N18, N12), eGo (E18, E12, E0)] aerosol and mainstream tobacco smoke (MS) extracts were prepared as previously described^36^. E0 corresponds to an e-cigarette aerosol without nicotine (Table S1). Cells were plated overnight, and exposure was initiated the following day. E-cigarette or MS extracts were added directly to the culture media to the desired extract concentration (0–39 ng nicotine/ml), a range similar to that observed in the plasma of vapers^34^. Sterile HEPES buffered saline was used as the vehicle control (C). Media was replaced every other day with freshly diluted extracts. Cisplatin was reconstituted in 0.9% w/v sodium chloride to a stock concentration of 1 mg/ml, and aliquots were kept at − 20 °C. For cell culture treatment, cisplatin was diluted to desired concentrations by serial dilution in complete media. 0.9% sodium chloride was used as a vehicle control. The amount of nicotine across extractions was measured by mass spectrometry.

### Cell proliferation/viability

Cells were plated at optimized numbers in 96-well plates and subsequently treated with e-cigarette aerosol extracts for 48 h. At 48 h, plates were replenished with media treated with e-cigarette extracts or treated with e-cigarette extracts and cisplatin. Cell viability was evaluated by the MTT (3-[4,5-dimethylthiazol-2-yl]-2,5-diphenyltetrazolium bromide) assay (Sigma Aldrich, Missouri, USA). 5 mg/ml MTT was added to cells and incubated for 4 h. The resultant formazan crystals were dissolved in the solvent (20% SDS, 50% DMF) overnight. MTT assay was performed at 96 h of e-cigarette exposure. OD values were obtained by spectrophotometry (BioTek Synergy H1 microplate reader, Vermont, USA). Experiments were repeated at least three times, each with six replicates.

### Cisplatin chemosensitivity assay

To examine cisplatin chemosensitivity, we measured cell viability using the MTT assay. In brief, cells were seeded in 96-well plates using optimized cell numbers for each cell line. Cells were incubated at standard conditions for 48 h with e-cigarette extracts, followed by treatment with e-cigarette extracts and cisplatin at concentrations (0.01, 0.1, 1, 10, 100 µM) for another 48 h. Cell viability was evaluated by the MTT as described above. The dose of cisplatin required to induce a 50% reduction in cell growth (the half-maximal inhibitory concentration, IC50) was computed from fitting a symmetrical sigmoidal curve to the logarithm of the response with the logarithm of the cisplatin concentration as the predictor using GraphPad Prism software, California, USA. Experiments were repeated at least three times, each with six replicates.

### Clonogenic survival assay

To assess colony survival, cell lines were exposed to e-cigarette extracts for 48 h, followed by another 48 h with e-cigarette extracts plus 2.5 μM cisplatin doses. Cells were trypsinized, counted, and seeded (at pre-optimized numbers determined from a test experiment using various cell dilutions to yield a trustable number of colonies) in six-well plates and assessed for colony formation. Cells were cultured without added extracts for 8–16 days. Media was replaced every three days to allow colony formation. Each experiment was performed three times with three technical replicates. At suitable time points, colonies were fixed with methanol followed by staining with 0.5% crystal violet in 25% methanol for 10 min. Colonies were washed with DI water and air-dried. Colonies with ≥ 50 cells were counted. Colony images were obtained using colony counter, Optronix GelCount, Abingdon, OX14 4SA, UK.

### Trypan blue exclusion assay

Cells were seeded in 48-well plates overnight. Cells were subsequently exposed to e-cigarette aerosol extracts for 48 h followed by treatment with e-cigarette aerosol extracts and 2.5 µM cisplatin for another 48 h before analysis. To assess cell death, cells were harvested using trypsin, pelleted, and stained with 0.4% trypan blue staining at a 1:1 ratio (v/v) and counted using a Countess II automated cell counter (Invitrogen) to determine the percentage of live and dead cells. Floating cells were included.

### RNA isolation and real-time PCR

Total RNA from cells exposed to extracts for 48 h was extracted using TRIzol reagent (Invitrogen) and quantified using a nano-drop followed by Reverse Transcriptase PCR using random primers per manufacturer’s recommendations. The 48 h time interval has been used as standard in the tobacco field to assess the effect of tobacco exposure in diverse cellular mechanisms including cisplatin resistance^35^. To determine the levels of mRNA expression, the first-strand cDNA was amplified using KAPA SYBR FAST Universal (KAPA Biosystems) on a BioRad CFX384 Real-Time System, BioRad, California, USA. The sequences of primers used are listed in Supplementary Table S2. β-actin was used as an endogenous control. Two to 3 experiments with 6 replicates each.

### Western blot analysis

Cells were exposed to e-cigarette aerosol extracts and MS smoke extracts for 48 h. Cells were trypsinized, pelleted, and washed with ice-cold phosphate-buffered saline (PBS) followed by resuspension in radioimmunoprecipitation assay (RIPA) buffer supplemented with 1X Protein Inhibitor Cocktail and phenylmethylsulfonyl fluoride (PMSF) (Roche). Protein concentration was measured using the Bio-Rad protein assay dye reagent concentrate (Cat. #500-0006) with bovine serum albumin (BSA) as the standard. 30–50 µg of protein per lane were loaded onto 7.5–10% sodium dodecyl sulfate–polyacrylamide gel electrophoresis (SDS-PAGE) and transferred to polyvinylidene difluoride (PVDF) membranes (Millipore, Massachusetts, USA). After blocking with 5% non-fat milk or 5% BSA, blots were incubated with primary antibodies overnight at 4 °C. The following primary antibodies were used: anti-ATP7A (Santa Cruz Biotechnologies, Texas, USA, sc-376467), anti-CTR1 (abcam, Massachusetts, USA, #ab129067), anti-ABCG2 (Cell Signaling Technology, Massachusetts, USA, #42078), and β-actin as a loading control (Cell Signaling Technology, Massachusetts, USA, #8457). Membranes were washed three times with Tris-buffered saline with 0.05% Tween and incubated in secondary antibodies for 1 h at room temperature. Secondary antibodies were rabbit anti-mouse IgG (abcam, Massachusetts, USA, #ab6728) and goat anti-Rabbit IgG (Cell Signaling Technology, Massachusetts, USA, #7074). The protein bands were visualized using the Clarity Western Blot enhanced chemiluminescence (ECL) Substrates (BioRad, BioRad, California, USA). Expression was detected using the ChemiDoc™ touch imaging system (BioRad, California, USA) and analyzed using Image Lab software (BioRad, California, USA).

### Statistical analysis

All data are expressed as mean ± SD unless otherwise stated. The difference in the treatment outcome (cell death, cell viability, colony survival, gene expression, and protein expression) by treatment (exposure to C, N12, N18, E0, E12, E18, or MS) was tested using One Way ANOVA, separately for all three cell lines. Linear regression analysis was performed to assess the effect of each treatment (exposure) on each outcome (cell death, cell viability, colony survival, gene expression, and protein expression) adjusting for cell line specific effect. A *p* value < 0.05 was considered statistically significant throughout the analysis. The analysis was performed using SAS software. IC50 values were obtained by analyzing the data using GraphPad Prism 5.0 software.

## Supplementary Information

Supplementary Information

## Data Availability

The datasets used and/or analyzed during the current study are available from the corresponding author on reasonable request.
