# Supplementary material for: Electronic cigarette aerosols alter the expression of cisplatin transporters and increase drug resistance in oral cancer cells
Source: Sci Rep. 2021 Jan 19;11:1821. doi: 10.1038/s41598-021-81148-0 (PMC7815770; doi:10.1038/s41598-021-81148-0)
Supplement: Supplementary file 1 — Supplementary Information [file 41598_2021_81148_MOESM1_ESM.pdf]

Electronic cigarette aerosols alter the expression of cisplatin transporters and increase drug resistance in oral cancer cells

Jimmy Manyanga[1,2], Vengatesh Ganapathy[1], Célia Bouharati[1], Toral Mehta[1], Balaji Sadhasivam[1], Pawan Acharya[3], Daniel Zhao[3,4], and Lurdes Queimado*[1,2,4]

Departments of [1]Otolaryngology Head and Neck Surgery, [2]Cell Biology, and *[3]Biostatistics & Epidemiology, The University of Oklahoma Health Sciences Center, Oklahoma City OK, USA.*
[4]The Peggy and Charles Stephenson Cancer Center, The University of Oklahoma Health Sciences Center, Oklahoma City OK, USA.

*Corresponding author: Lurdes Queimado, MD, Ph.D., Department of Otolaryngology Head and Neck Surgery, The University of Oklahoma Health Sciences Center, 800 Research Parkway, Room 431, Oklahoma City, OK 73104. Phone: (405) 271-4232; Fax: (405) 271-9364. E-mail: lurdes-queimado@ouhsc.edu

Supplementary Figures: 4

Supplementary Tables: 2

Table S1

Table S1. Nicotine concentration in e-liquids and in exposure e-cigarette extracts.

E-cigarette	Nicotine in e-liquid# (mg/ml)	Nicotine in diluted e-cigarette extracts* (ng/ml)
N12	12	5.1
N18	18	39.1
E0	0	0
E12	12	10.7
E18	18	34.3

#Reported by manufacturer; *Measured by gas chromatography mass spectrometry (GCMS)

Figure S1

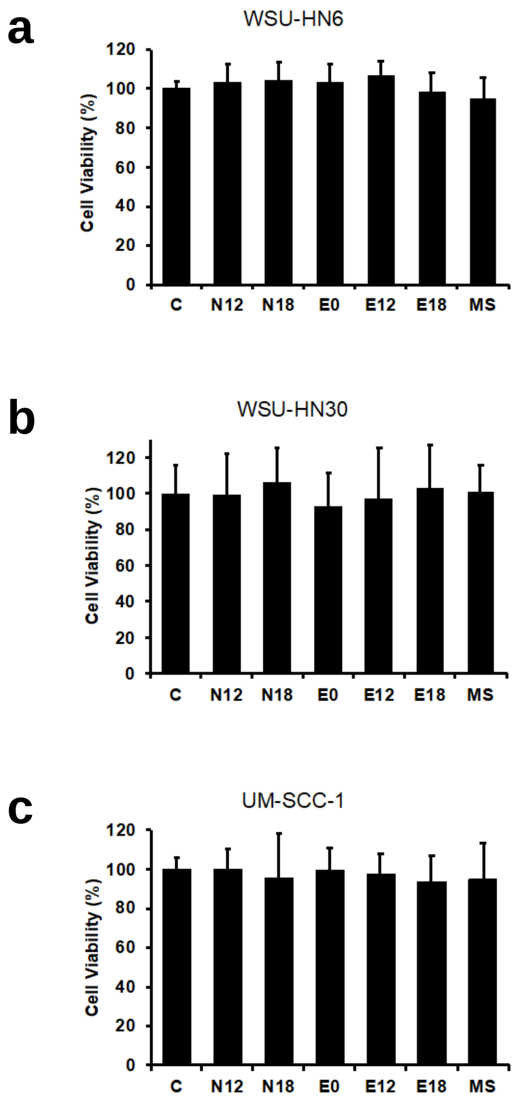

Figure S1. Exposure to e-cigarette aerosol alone does not alter cell proliferation. WSU-HN6 (**a**), WSU-HN30 (**b**), and UM-SCC-1 (**c**) cell lines were exposed to e-cigarette aerosol extracts for 96 h and viability assessed by MTT. No significant difference in cell proliferation between vehicle control and e-cigarette or MS extract-treated cells was observed across treatments for all cell lines.

Figure S2

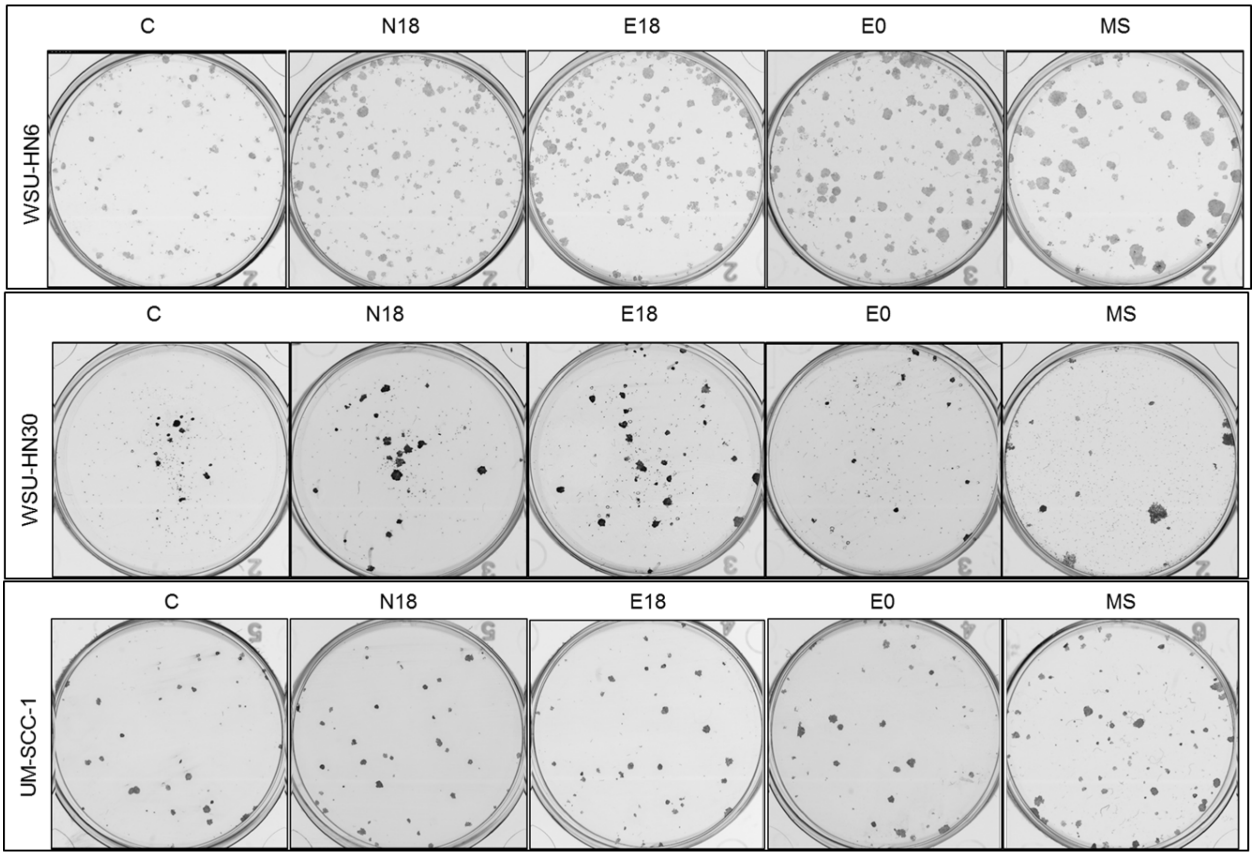

Figure S2. Exposure to e-cigarette increases clonogenic survival after cisplatin. Representative images of WSU-HN6, WSU-HN30, and UM-SCC-1 cell colonies formed 2 weeks after treatment with cisplatin in the presence and absence of extracts.

Figure S3

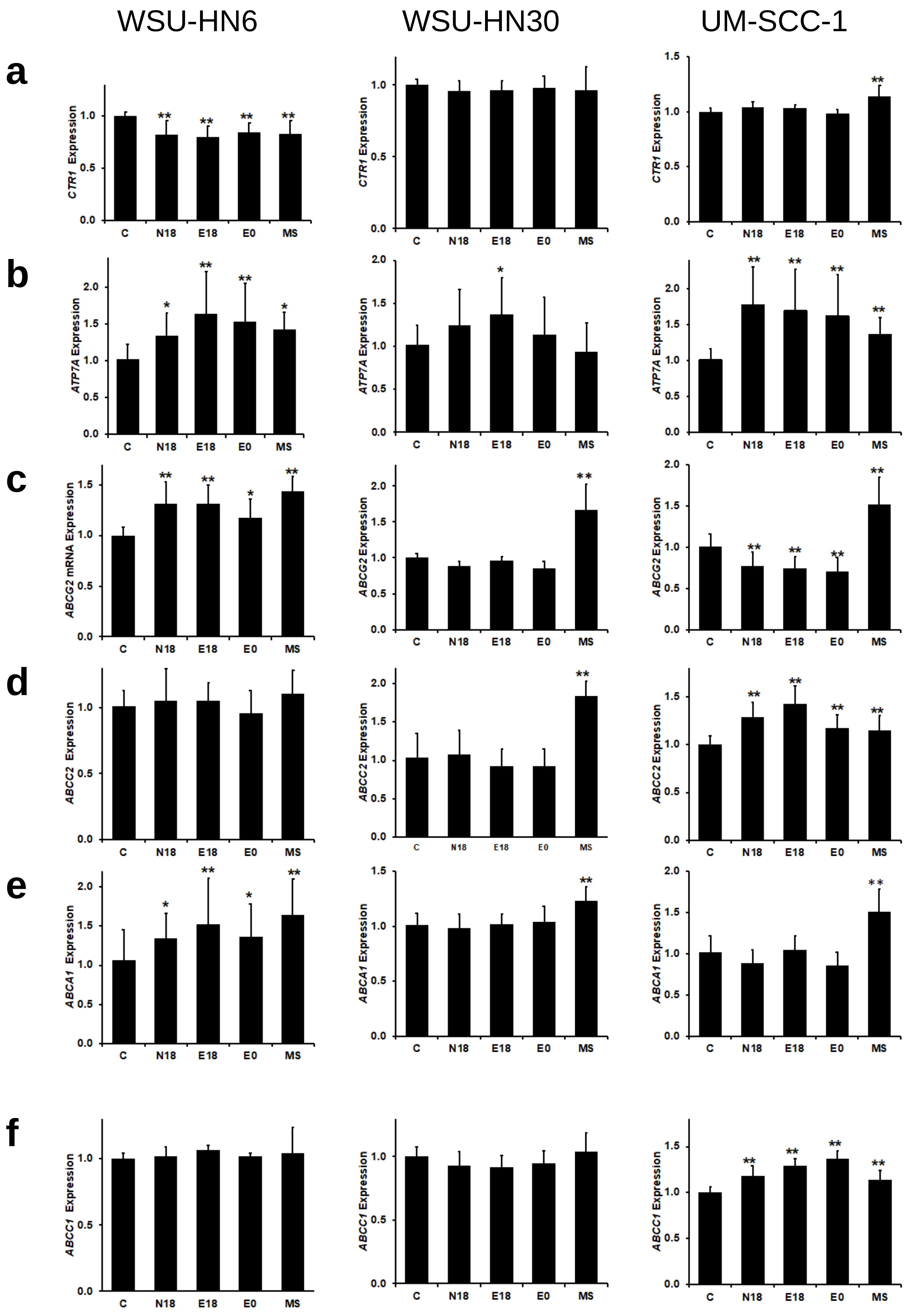

WSU-HN6
WSU-HN30
UM-SCC-1
a
b
c
d
e
f
CTR1 Expression
ATP7A Expression
ABCG2 mRNA Expression
ABCG2 Expression
ABCC2 Expression
ABCA1 Expression
ABCC1 Expression
C
N18
E18
E0
MS

g

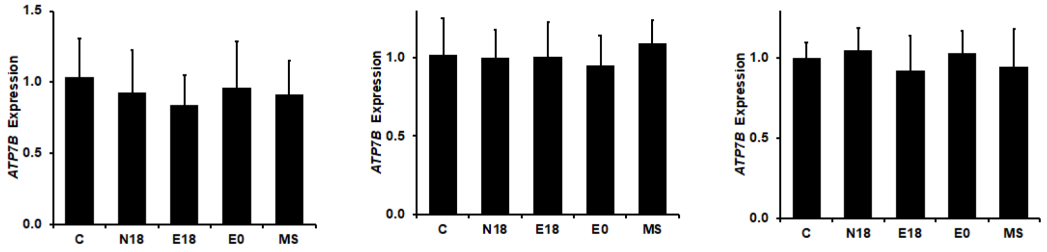

Figure S3. Exposure to e-cigarette aerosol extracts alters the expression of multidrug drug resistance genes. (**a**) *CTR1*, (**b**) *ATP7A*, (**c**) *ABCG2*, (**d**) *ABCC2*, (**e**) *ABCC1*, (**f**) *ABCA1* and (**g**) *ATP7B* mRNA expression from oral cancer cell lines exposed to e-cigarette aerosol extracts for 48 h was analyzed by qRT-PCR. The expression of multidrug-resistant genes after e-cigarette extract exposure varied by cell line for individual genes. *p<0.05, **p<0.001 by one-way ANOVA.

Table S2

Table S2. Primer sequences used for RT-PCR.

Primer	Forward	Reverse
β-actin	5'-GTCATCACCATTGGCAATGAG-3'	5'-ATGTCCACGTCACACTTCATG-3'
ATP7A	5'-GCAGAGCCTCTATAAACTCAC-3’	5’-GTGTCATCATCTTCCCTGAAG-3'
ATP7B	5'-GACCAGGTCAGCTATGTCAG-3’	5’-CATCAGATGTACTGCTCCTCA-3'
hCTR1	5'-CTCATCTTCATGACCTACAACG-3’	5’-GATGTCAATGGCAATGCTCTG-3'
ABCG2	5'-TGTGGCATTAAACAGAGAAGAAGAC-3'	5'-TCACCCCCGGAAAGTTGATG-3'
ABCA1	5'-CTCAGACAACACTTGACCAAG-3’	5’-AGATGTGAGAACTGCAACGTC-3'
ABCC1	5'-ACACAGTTCGAGGACTGCAC-3’	5’-GAAAAGACCTCTCTGCTGCAG-3'
ABCC2	5'-AGGTAATGGTCCTAGACAACG-3’	5’-GTGCTGTTCACATTCTCAATGC-3'
ERCC1	5’ -GGCGACGTAATTCCCGACT-3’	5’ -TAGCGGAGGCTGAGGAACA-3’
XPA	5'-GCAGCCCCAAAGATAATTGA-3'	5'-TGGCAAATCAAAGTGGTTCA-3'
MMS19	5'-GTCAGCAGGACCCTGAGAGTTC-3'	5'-CTCAGAACTGAGGGCTCCTTC-3'

Figure S4

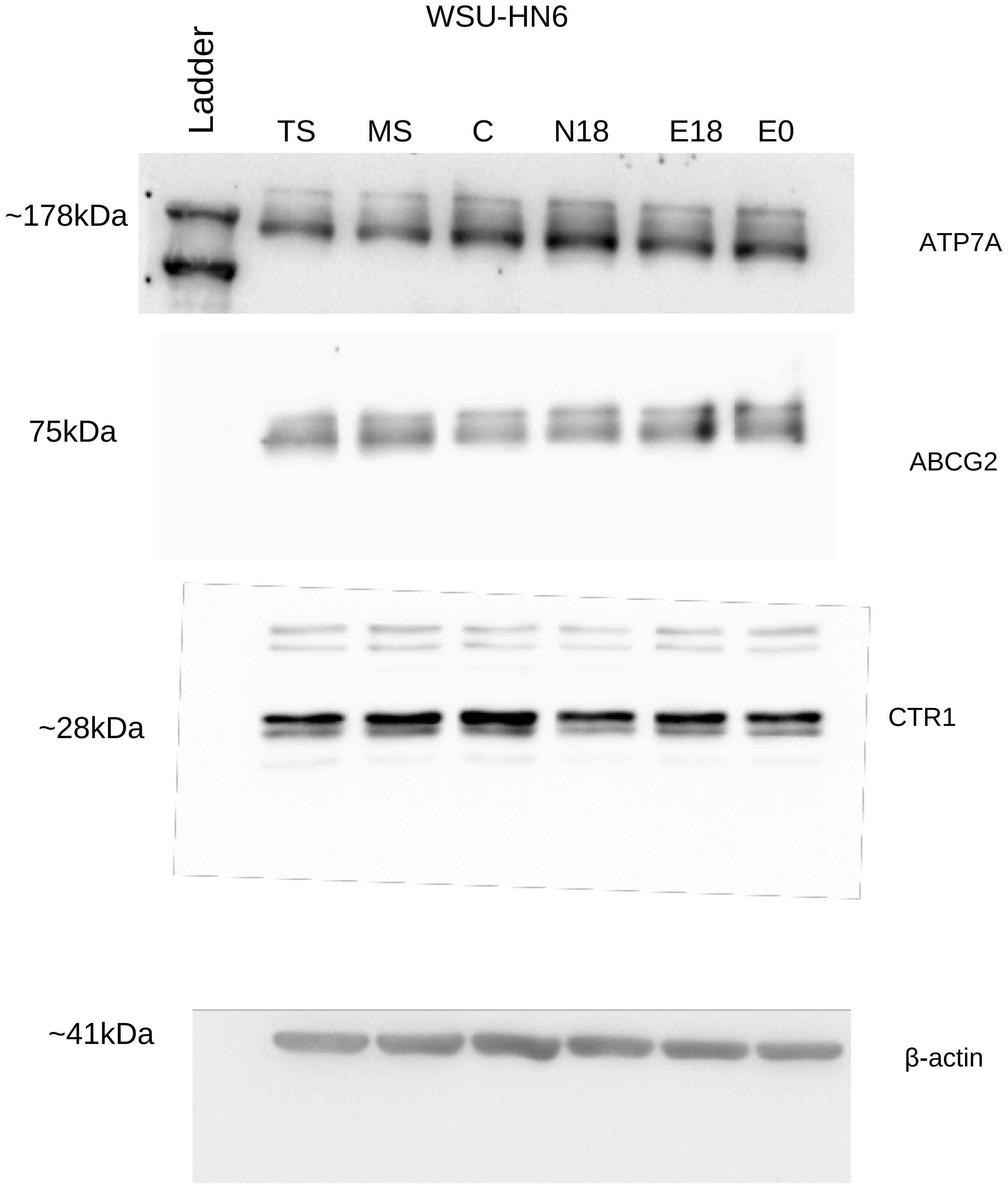
WSU-HN6
Ladder
TS
MS
C
N18
E18
E0
~178kDa
ATP7A
75kDa
ABCG2
~28kDa
CTR1
~41kDa
β-actin

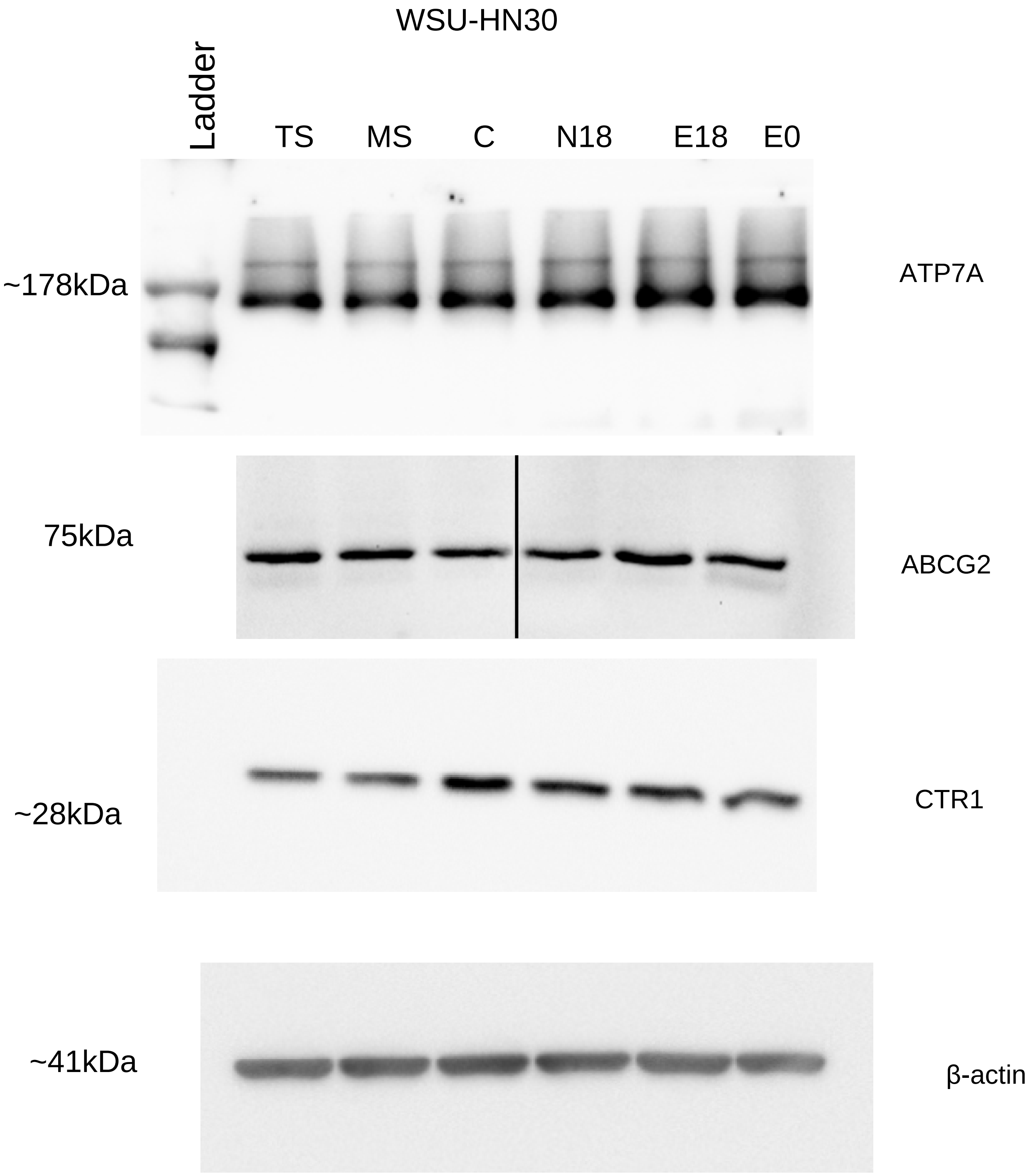
WSU-HN30
Ladder
TS
MS
C
N18
E18
E0
~178kDa
ATP7A
75kDa
ABCG2
~28kDa
CTR1
~41kDa
β-actin

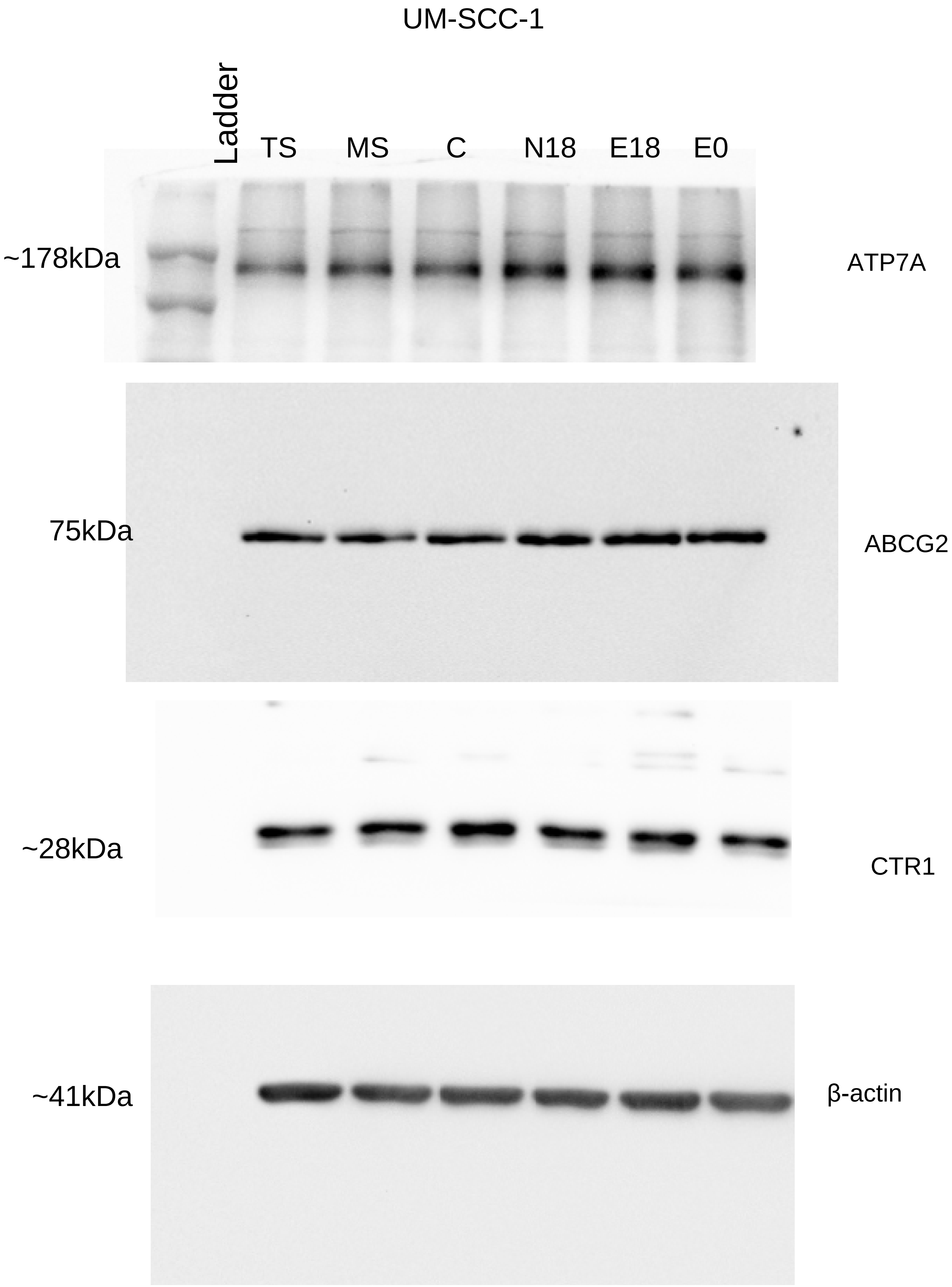

Figure S4. Full original, unprocessed blots used for figure 5. Membranes were cut into strips before hybridization of indicated proteins. (0-40 kDa to detect CTR1; 40-55 kDa for B-

actin; 55-100 kDa for ABCG2; and 100-250 kDa to detect ATP7A). TS represents tobacco smoke. Vertical line delineates lane re-arrangement for the ABCG2 blot.
